# Daily temporal dynamics of vaginal microbiota before, during and after episodes of bacterial vaginosis

**DOI:** 10.1186/2049-2618-1-29

**Published:** 2013-12-02

**Authors:** Jacques Ravel, Rebecca M Brotman, Pawel Gajer, Bing Ma, Melissa Nandy, Douglas W Fadrosh, Joyce Sakamoto, Sara SK Koenig, Li Fu, Xia Zhou, Roxana J Hickey, Jane R Schwebke, Larry J Forney

**Affiliations:** 1 Institute for Genome Sciences, University of Maryland School of Medicine, 801 W Baltimore Street, Baltimore, MD 21201, USA; 2 Department of Microbiology and Immunology, University of Maryland School of Medicine, 685 West Baltimore Street, HSF-I Suite 380, Baltimore, MD 21201, USA; 3 Department of Epidemiology and Public Health, University of Maryland School of Medicine, 660 W. Redwood Street, Howard Hall Suite 200, Baltimore, MD 21201, USA; 4 Department of Biological Sciences, University of Idaho, Life Sciences South 252, 875 Perimeter Drive MS 3051, Moscow, ID 83844, USA; 5 Institute for Bioinformatics and Evolutionary Studies (IBEST), University of Idaho, 875 Perimeter Drive MS 3051, Moscow, ID 83844, USA; 6 Department of Medicine, University of Alabama at Birmingham, 1808 7th Ave S, Birmingham, AL 35294, USA

## Abstract

**Background:**

Bacterial vaginosis (BV) is a common gynecologic diagnosis characterized by dysbiosis of the vaginal microbiota. It is often accompanied by vaginal symptoms such as odor and discharge, but can be asymptomatic. Despite over 50 years of research, the etiology of BV is not well understood, which is a major impediment to treatment and prevention of BV.

**Results:**

Here we report on the temporal dynamics of 25 vaginal communities over a 10 week period using samples collected daily from women who were diagnosed with symptomatic BV (15 women), asymptomatic BV (6 women), and women who did not have BV (4 women).

**Conclusion:**

This unique resource of samples and data will contribute to a better understanding of the role that the vaginal microbes have in the natural history of BV and lead to improved diagnosis and treatment.

## Background

Bacterial vaginosis (BV) is the most common vaginal condition of women of reproductive age, resulting in millions of healthcare visits annually in the United States alone [1]. Women with BV typically report symptoms that include a thin vaginal discharge and a fishy malodor [2]; however, a substantial portion of affected women are asymptomatic [3]. Although the etiology of BV is incompletely understood, it is well-documented that BV is accompanied by, and perhaps caused by, disruption of the vaginal ecosystem that is reflected in alterations to the composition and structure of vaginal microbial communities [4,5]. In women with BV, there are reduced proportions of lactic acid–producing bacteria (primarily *Lactobacillus* spp.) and increases in the number and diversity of facultative and strictly anaerobic bacteria, including species of *Gardnerella*, *Prevotella* and other taxa of the order Clostridiales [4]. BV has been shown to be an independent risk factor for adverse outcomes, including preterm delivery and low infant birth weight, the development of pelvic inflammatory disease [6] and increased risk of acquiring sexually transmitted infections and HIV [7-10]. National surveys estimate that the prevalence of BV among US women is 29% [11], and, despite the high burden of this disease, the events that precipitate BV remain obscure. BV is particularly troubling for patients and clinicians, as recurrence of BV following antibiotic treatment is common [12] and the arsenal of treatment includes just two antibiotics, namely, topical or oral metronidazole and clindamycin [2].

In clinical settings, BV is often diagnosed based on the criteria described by Amsel *et al*. [13], wherein three of the following four signs must be evident: (1) a homogeneous, white, noninflammatory discharge that smoothly coats the vaginal walls; (2) the presence of clue cells (squamous epithelial cells covered with adherent bacteria) upon microscopic examination; (3) a vaginal fluid pH over 4.5; and (4) a fishy odor after addition of 10% KOH to vaginal secretion samples. In research and laboratory settings, BV has also been diagnosed by scoring a Gram-stained vaginal smear [14-16]. One scoring method described by Nugent *et al.*[14] reflects the relative abundance of large Gram-positive rods (lactobacilli), Gram-negative and Gram-variable rods and cocci (including, *G. vaginalis*, *Prevotella*, *Porphyromonas* and peptostreptococci) and curved Gram-negative rods (*Mobiluncus*). The relative proportions of these different bacterial morphotypes give rise to a score that ranges from 0 to 10. A score of 0 to 3 is considered healthy, 4 to 6 is intermediate and 7 to 10 is indicative of BV. A weakness of diagnostic tests based on Gram-stained smears is the subjective nature of assessing bacterial cell morphology [17]. Gram staining can be performed on self-collected vaginal smears [18], which is critical for the success of longitudinal, field-based studies [19,20].

Previous studies of BV have focused largely on women who presented to their physicians with symptoms or cohorts of women who were treated for BV and followed for extended observational periods with infrequent sampling. In one such study, Srinivasan *et al.* followed women diagnosed with BV daily for 7 days, then at 2, 3 and 4 weeks, and used quantitative PCR to determine the abundance of specific populations in vaginal samples [21]. The study demonstrated that vaginal microbiota are dynamic and that antibiotic treatment for BV rapidly reduces the number of facultative and strictly anaerobic species, but these bacteria later reemerged. These findings are consistent with the well-known phenomena of recurrent BV, in which the signs and symptoms of BV are alleviated by antibiotic use but return in the days and weeks that follow a course of therapy. These patterns of recurrence suggest that the vaginal environments of these women select for various populations of anaerobes. A better understanding of the events and conditions that cause shifts in the composition of the vaginal microbiome is needed to devise therapies that reduce the incidence of BV and its recurrence following antibiotic therapy.

In this study, we sought to evaluate the spectrum of events that occur in vaginal microbial communities prior to, during and after episodes of BV by characterizing the composition and dynamics of vaginal bacterial communities using high-throughput 454 pyrosequencing of barcoded V1 to V3 regions of 16S rRNA genes. To do this, we conducted a high-resolution prospective study in which samples were collected daily from 135 women of reproductive age over two menstrual cycles. Behaviors and events that took place before, during and after BV episodes were recorded. Herein we report our initial findings on the daily composition and relative abundance of bacteria in vaginal samples from 25 women, 15 of whom experienced symptomatic BV (SBV), 6 who were diagnosed with asymptomatic BV (ABV) and 4 who remained healthy during the 10-week study. These women were selected on the basis of their clinical examinations and the longitudinal patterns of changes in Nugent scores.

## Methods

### Participants and sample collection

Between September 2009 and July 2010, 135 nonpregnant women of reproductive age were enrolled in a longitudinal study at the University of Alabama at Birmingham. The clinical study protocol was approved by the Institutional Review Board of the University of Alabama at Birmingham and the University of Maryland School of Medicine. Written informed consent was appropriately obtained from all participants.

At study entry, participants were asked to answer a lengthy questionnaire on demographics as well as medical, dental, obstetric, hygiene, sexual and behavioral histories. They were also asked to self-collect three midvaginal swabs daily for ten weeks. The first Copan ESwab (Copan Diagnostics, Murrieta, CA, USA) was placed in RNA*later* (Ambion, Austin, TX, USA) for use in future metatranscriptomics analyses. A second Copan ESwab was placed in Amies liquid transport medium for use later in extracting genomic DNA. Third, a Starplex double-headed Dacron swab (Starplex Scientific, Cleveland, TN, USA) was stored dry in a tube for later use in metabolomic and metaproteomic analyses. One of the Dacron swabs was also used to prepare a smear that was later Gram-stained for Nugent scoring. In addition, the participants measured their vaginal pH using the CarePlan VpH test glove (Inverness Medical Innovations, Waltham, MA, USA). Finally, participants completed a diary each day to record hygienic practices and sexual activities using a standardized form on which all responses were precoded. Pelvic examinations were performed at the time of enrollment and at weeks 5 and 10 or at interim times if the participant reported vaginal symptoms. A diagnosis of SBV was made when three of four Amsel criteria were recorded by the clinician and the participants reported symptoms spontaneously or upon direct questioning. ABV was defined as a finding of three of four Amsel criteria, but without self-report of any vaginal symptoms. Women diagnosed with SBV were treated for BV using standard of care practices [22]. Women with ABV were not treated. Clinical and selected daily behavioral data are described in Additional file 1.

### DNA extraction and purification

Genomic DNA was extracted from vaginal swabs that had been stored in Amies liquid transport medium at -80°C. We used a validated procedure [23-25] that includes steps for enzymatic and physical lysis of bacterial cells followed by purification of genomic DNA using a QIAsymphony robotic platform and QIAGEN CellFree 500 kits (QIAGEN, Valencia, CA, USA) according to the manufacturer’s protocol. This procedure provided between 2.5 and 5 μg of high-quality genomic DNA from 300 μl of each sample resuspended from a vaginal swab (Additional file 2).

Polymerase chain reaction amplification and sequencing of the V1 to V3 region of bacterial 16S rRNA genes.

The composition and abundance in vaginal bacterial communities was determined using culture-independent methods. The V1 to V3 hypervariable region of 16S rRNA genes was amplified using an optimized barcoded primer set targeting 27f [26] and 533r. Amplifications were performed in 96-well plates using the HotStar HiFidelity DNA Polymerase Kit (QIAGEN). Amplicons were quantified using the Quant-iT PicoGreen kit (Molecular Probes/Invitrogen, Eugene, OR, USA), then equimolar amounts (100 ng) of amplicons were mixed in a single tube prior to purification with the Agencourt AMPure XP PCR Purification System (Beckman Coulter, Brea, CA, USA). Purified amplicon mixtures were sequenced by 454 pyrosequencing (454 Life Sciences, Branford, CT, USA) at the Genomics Resource Center of the Institute for Genome Sciences, University of Maryland School of Medicine.

## Quality assurance

### Sequence reads quality control, analysis and taxonomic assignments

Individual reads were quality-checked to eliminate short or chimeric reads, reads with ambiguous base pairs and those with low-quality regions using the criteria described in the Supplementary methods section. Taxonomic assignments were performed using a combination of a phylogenetics-based classifier and speciateIT software (http://www.speciateIT.sourceforge.net) (Additional file 3).

## Initial findings

A total of 1,657 samples (a mean of 66.3 per woman, and a median of 67 per woman) were successfully sequenced, and 8,757,681 high-quality sequenced reads were generated with a mean of 5,285 and median of 5,093 reads per sample. Lower read counts were obtained for samples from subject 3 and subject 5 (weeks 1 to 3); however, these samples are still comparable to those with higher read counts as demonstrated previously [27] and are included in this data set. The mean read length was 485 bp (median of 515 bp).

**Figure 1:**
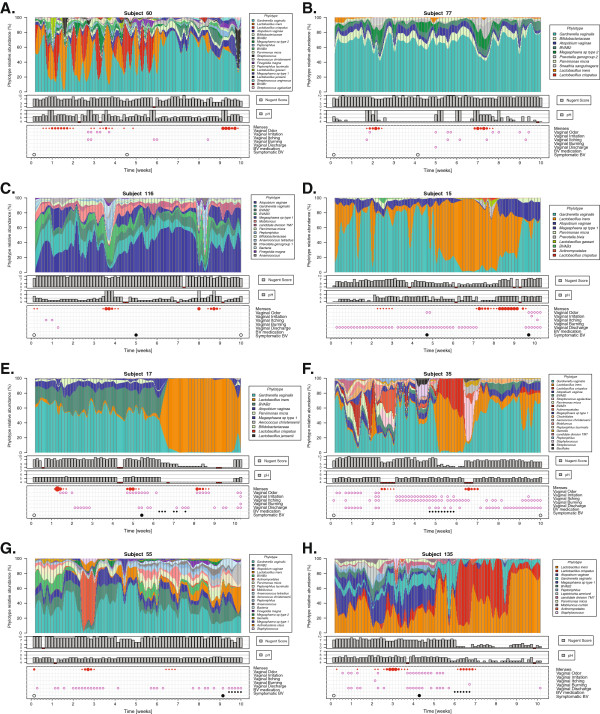
**Daily temporal dynamics of vaginal bacterial communities. (A)** and **(B)** Women diagnosed with asymptomatic bacterial vaginosis (ABV) at some point during the ten-week period are shown. **(C)** to **(H)** women diagnosed with symptomatic bacterial vaginosis (SBV) at some point during the ten-week period are shown. **(A)** through **(H)** Color codes for each phylotype represented in the interpolated bar plots are shown next to each panel. Pink open circles indicate symptomology. Red closed circles indicate menstruation days. Large black open circles represent ABV diagnosis. Large black closed circles represent symptomatic BV diagnosis. Small closed circles indicate BV medications used. Daily Nugent scores (range: 0 to 10) and pH (range: 4 to 7) are indicated below the graphs. See Additional file 4: Figure S1 for interpolated bar plots for all subjects.

Unexpectedly, the vaginal microbiota prior to SBV mainly comprised strict anaerobes, such as *Atopobium*, *Prevotella*, *Megasphaera*, BV-associated bacterium 2 and the facultative anaerobe *G. vaginalis*, and the vaginal pH was elevated (>4.5) (Figure 1). During the 2- to 9-week interval prior to the diagnosis of SBV, a few symptoms were reported in daily diaries, but none of these prompted participants to immediately seek medical attention. Women who were diagnosed with ABV harbored vaginal microbiota that lacked significant proportions of *Lactobacillus* spp. and had symptoms similar to those of patients who ultimately were diagnosed with SBV (Figure 1A and B). Of note, *Lactobacillus iners* was consistently present in women who had SBV or ABV, albeit in low proportions (Figure 1 and Additional file 4: Figure S1). In most women, the treatment of SBV reduced the proportion of facultative and strict anaerobes and increased the relative proportions of *Lactobacillus* spp. (mainly *L. iners*) (Figure 1D to F). This effect was short-lived, however, and, in most individuals, the community returned to its pretreatment state within 2 to 4 weeks. Community dynamics in women who had ABV or SBV appeared to be highly personalized, with some women experiencing rapid shifts in community composition (Figure 1A, F and G) and others harboring stable, but *Lactobacillus* spp.-depleted, microbiota (Figure 1B to E and H). The vaginal microbiota of women who did not have SBV or ABV were consistently dominated by *Lactobacillus* spp. or *Bifidobacterium*, but were not always stable in terms of the dominant species of *Lactobacillus* present.

## Future directions

We will use these data to examine shifts in vaginal microbial community composition in conjunction with epidemiological and behavioral data to better define BV and identify patterns that predict an increased risk for the disease. This study will be done using model-based statistical clustering and classification approaches to associate microbial community composition and dynamics with metadata and clinical diagnoses of health or BV. The large body of information generated will facilitate understanding vaginal microbial community dynamics and the etiology of BV, and it will drive the development of better diagnostic tools for use in the differential diagnosis of BV. Furthermore, we anticipate that the information will enable a more personalized, more effective treatment of BV and ultimately help to prevent adverse sequelae associated with this highly prevalent disruption of the vaginal microbiome.

## Supporting data

All sequence data and metadata were deposited in the Sequence Read Archive (SRA; http://www.ncbi.nlm.nih.gov/Traces/sra/) under BioProject PRJNA208535 (“The daily dynamics of the vaginal microbiota before and after bacterial vaginosis”; http://www.ncbi.nlm.nih.gov/bioproject/?term=PRJNA208535) ([SRP026107] and [SRA091234]). Quantitative Insights Into Microbial Ecology (QIIME software) mapping files are provided in Additional file 5. Because of the sensitivity of the metadata and Institutional Review Board restrictions, additional metadata can be requested only directly from the principal investigators.

## Abbreviations

PCR: Polymerase chain reaction.

## Competing interests

The authors declare that they have no competing interests.

## Authors’ contributions

JR, RMB, PG and LJF designed the study. JRS lead the clinical study and sample collection. MN, DWF, JS, SSKK, LF and XZ processed the samples and generated the sequence data. PG, BM, JR, XZ, RJH and LJF analyzed and interpreted the data. JR, PG, RMB and LJF wrote the manuscript. All authors read and approved the final manuscript.

## Supplementary Material

Additional file 1 Clinical and daily behavioral data. Click here for file

Additional file 2 Supplementary methods. Click here for file

Additional file 3 Relative proportions of taxa observed in all samples. Click here for file

Additional file 4: Figure S1 Daily temporal dynamics of vaginal bacterial communities in 15 women who experienced symptomatic BV diagnosis (A to O), asymptomatic BV only (P to U) at some point during a 10-week period or no BV (V to Y). Color codes for each phylotype represented in the interpolated bar plots are shown next to each panel. Pink open circles: symptomology; red closed circles: menstruation days (small circles indicate low bleeding, medium circles, medium bleeding and large circles heavy bleeding); large black open circles: asymptomatic BV diagnosis; large black closed circles: symptomatic BV diagnosis; small closed circles: BV medication used. Daily Nugent scores (range 0–10) and pH (range 4–7) are indicated below the graph. Click here for file

Additional file 5 QIIME mapping files for each 454 sequencing run. Click here for file
