# Supplementary material for: Daily temporal dynamics of vaginal microbiota before, during and after episodes of bacterial vaginosis
Source: Microbiome. 2013 Dec 2;1:29. doi: 10.1186/2049-2618-1-29 (PMC3968321; doi:10.1186/2049-2618-1-29)
Supplement: Additional file 2 — Supplementary methods. [file 2049-2618-1-29-S2.docx]

**Supplemental Materials**

**Daily temporal dynamics of vaginal microbiota before, during and after episodes of bacterial vaginosis**

Ravel *et al*. Microbiome

**Experimental design, sampling and sample storage**

Non-pregnant adult women between the ages of 18 and 45 were recruited to participate in a longitudinal study with clinical visits at baseline, week 5 and week 10. Pregnancy was an exclusion criterion. At baseline, participants collected a urine sample for pregnancy testing based on beta human chorionic gonadotropin (hCG). During the course of the longitudinal follow-up, participants with more than 35 days since last menstrual cycle were asked to return for urine hCG pregnancy testing. The remaining urine was stored in a freezer archive. Study personnel also collected a blood sample for herpes simplex virus (HSV) type 1 & 2 screening at baseline. HSV was not an exclusion criteria but the information will be important in modeling the vaginal microbiome. The remainder of blood was stored in a freezer archive.

At each clinical visit, a research nurse administered a questionnaire which collected information on socioeconomic and demographic factors, feminine hygiene practices and health behaviors, gynecological and obstetrical history, sexual history and practices, sexually transmitted infection history, date of last menstrual period, methods of birth control used, alcohol and drug use, fitness status and dental history. The research nurse also assessed pelvic symptoms, performed a limited physical examination, collected vaginal biological specimens (see below), recorded any physical findings including vaginal discharge and easily induced bleeding, and assessed the occurrence of ectopy, edema, inflammation, or ulcerations. During the pelvic examination, the nurse collected materials for the clinical assessment of bacterial vaginosis (BV) using the Amsel [[1](#_ENREF_1)] and Nugent criteria [[2](#_ENREF_2)]. The research nurse collected two additional swabs during the gynecological and pelvic exams. One endocervical swab was used for the screening of *Neisseria gonorrhea* (GC) and *Chlamydia trachomatis* (CT) by nucleic acid amplification tests (Becton Dickinson, Sparks, MD, BD ProbeTec ET)*.* One vaginal swab was used for *Trichomonas vaginalis* (TV) screening (inPouch). If a participant tested positive for GC or CT, study personnel reported the participant’s test results to the health department as required by law. These participants were offered treatment or a prescription for treatment at the time they were notified of the test results and permitted to continue in the study. If a participant was diagnosed by clinical exam or laboratory screening with other treatable conditions including mucopurulent cervicitis, bacterial vaginosis (symptomatic), candidiasis (symptomatic), or trichomoniasis the treatment or a prescription for treatment was provided. If the diagnosis was made at baseline, participants were offered enrollment 30 days after treatment was completed. If the diagnosis was made during the study observation, the participant continued in the study without interruption.

Participants known to be HIV-positive were excluded from the study. Medical record information, including HIV and syphilis screening, were available from participants if they had been screened at the Jefferson County Department of Health (JCDH) where HIV-testing is performed by ELISA and presumptive positive results are confirmed by Western Blot.

Subjects were asked to self-collect vaginal swab samples daily for 10 weeks. At the baseline visit, participants were given the materials needed for one week of vaginal self-sampling. They were also provided detailed instructions on procedures to be used for the self-collection of vaginal swabs, preparation of vaginal smears, and instructions for swab storage and transport back to the clinic. On a daily basis each subject self-collected three mid-vaginal vaginal swabs: the first Copan E-Swab was placed in RNAlater (Ambion) for use in future metatranscriptomics analyses; a second Copan E-Swab was placed in Liquid Amies Transport Media for use later in extracting genomic DNA; and a Dacron Starplex double headed swab. The latter swab was used to prepare a smear that was later Gram stained, and the swabs were stored dry in a tube for later use in metabolomic and metaproteomic analyses. In addition, subjects measured vaginal pH using the CarePlan® VpH test glove (Inverness Medical). Finally, a diary was completed each day using a standardized form on which all responses were pre-coded to record hygiene practices and sexual activities. The diary included information on the use of sanitary napkins, tampons, and douching, as well as vaginal intercourse, receptive oral sex, digital penetration, rectal sex, sex toys or the use of diaphragms, condoms, spermicides, lubricants. Women also reported menstrual bleeding, and vaginal symptoms (vaginal itching, discharge, odor, irritation and pain on urination).

After all samples were collected they were stored in the participants’ freezers. Each week the subjects transported their samples in a cooler to the study site where they were then transferred to a -80°C freezer. At this time another one-week sampling kit was provided to the study subjects. If a participant consistently reported discomfort or vaginal irritation with the use of the Copan e-swabs, she was switched to collect samples with a Dacron swab (Starplex), which was also stored in Liquid Amies Transport Media and RNAlater.

All samples were overnight shipped on dry ice to the University of Maryland School of Medicine for storage and processing. Integrity of samples were checked upon receipt and the shipping manifest compared against the study FreezerWorks database prior to archiving.

All vaginal smears from daily sampling were Gram-stained and scored using Nugent criteria at the University of Alabama at Birmingham [[2](#_ENREF_2)]. Nugent scores are composite scores based on the cellular morphologies of the bacteria present in a sample. A score of 0-3 was designated normal, 4-6 as intermediate and 7-10 was considered to be abnormal and indicative of bacterial vaginosis. Over 9,000 slides were scored.

Batches of samples were shipped to the Institute for Genome Sciences at weekly intervals whereupon the samples were again stored at -80°C. In total over 33,000 biological samples were collected in this study. All data from this study are managed and stored at the Institute for Genome Sciences at the University of Maryland School of Medicine in a secure relational database that includes all de-identified metadata (medical evaluations, answers to all questionnaires, and daily diaries) and a system to track barcoded samples from each participant.

**Nucleic acid isolation**

Genomic DNA was extracted from vaginal swabs stored in Amies transport media and stored at -80°C. Procedures for the extraction of genomic DNA from frozen vaginal swabs have been developed and validated [[3](#_ENREF_3), [4](#_ENREF_4)]. Briefly, frozen vaginal swabs were immersed in 1 ml of pre-warmed (55°C) cell lysis buffer composed of 0.05M potassium phosphate buffer containing 50 µl lyzosyme (10 mg/ml), 6 µl of mutanolysin (25,000 U/ml; Sigma-Aldrich) and 3 µl of lysostaphin (4,00 U/ml in sodium acetate; Sigma-Aldrich) and the mixture was incubated for 1 hour at 37°C. Then 10 µl proteinase K (20 mg/ml), 100 µl 10% SDS, and 20 µl RNase A (20 mg/ml) were added and the mixture was incubated for 1h at 55ºC. The samples were transferred to a FastPrep Lysing Matrix B tube (Bio101) and microbial cells were lysed by mechanical disruption using a bead beater (FastPrep instrument, Qbiogene) set at 6.0 m/s for 30 sec. The lysate was processed using the CellFree500 kit on a QIAsymphony robotic platform. The DNA was eluted into 100 µl of TE buffer, pH 8.0. This procedure provided between 2.5 and 5 µg of high quality whole genomic DNA from vaginal swabs.

**PCR amplification and sequencing of the V1-V3 region of bacterial 16S rRNA genes**

The microbial species composition and abundance in vaginal communities was determined using culture-independent methods. The V1-V3 hypervariable regions of the 16S rRNA genes were amplified using an optimized primer set comprising 27F [[5](#_ENREF_5)] and 534R. Because primer 534R contains a unique sample identifying barcode, up to 192 samples were sequenced on one sequencing run and generated 4,000 to 6,000 sequence reads per sample. A total of 96 unique 534R primers each with a specific barcode were used. The primers were as follows:

27F - 5’-*GCCTTGCCAGCCCGCTCAGTC****AGAGTTTGATCCTGGCTCAG***-3’

534R - 5’-*GCCTCCCTCGCGCCATCAGNNNNNNNNCA****TTACCGCGGCTGCTGGCA-3’***

where the underlined sequences are the 454 Life Sciences® primers B and A in 27F and 534R, respectively, and the bold font denotes the universal 16S rRNA primers 27F and 534R. The barcode within 534R is denoted by 8 Ns but actually varies from 6 to 8Ns. These barcodes were identical to those used by the Human Microbiome Project [[6](#_ENREF_6)]. A mixture of bacterial 27F primers was used to maximize sequence type discovery and eliminate the PCR amplification bias described by Frank et al. [[5](#_ENREF_5)]. The 27F formulation remains relatively simple, having only seven distinct primer sequences so there is minimal loss of overall ampliﬁcation efﬁciency and speciﬁcity. The 27F primer mixture was: 27f-CM (5’-AGAGTTTGATCMTGGCTCAG, where M is A or C), four-fold degenerate primer 27f-YM (5’-AGAGTTTGATYMTGGCTCAG, where Y is C or T), or seven-fold degenerate primer 27f-YM+3. The seven-fold degenerate primer 27f-YM+3 is four parts 27f-YM, plus one part each of primers speciﬁc for the ampliﬁcation of Biﬁdobacteriaceae (27f-Bif, 5’-AGGGTTCGATTCTGGCTCAG), *Borrelia* (27f-Bor, 5’-AGAGTTTGATCCTGGCTTAG), and Chlamydiales (27f-Chl, 5’-AGAATTTGATCTTGGTTCAG) sequences. This primer formulation was previously shown to better maintain the original rRNA gene ratio of *Lactobacillus* spp. to *Gardnerella* spp. in quantitative PCR assays, particularly under stringent ampliﬁcation conditions [[5](#_ENREF_5)].

For every set of 192 vaginal genomic DNA samples PCR amplification of 16S rRNA genes was performed in 96-well microtiter plates as follows: 1X PCR buffer, 0.3 µM primer 27F and 534R, 0.25 µl HotStar HiFidelity DNA polymerase (5U/µl; Qiagen), and 25 ng of template DNA in a total reaction volume of 25 µl. Reactions were set up on a QIAgility robotic platform. Reactions were run in a DNA engine Tetrad2 instrument (Bio-Rad) using the following cycling parameters: 5 min denaturing at 95°C followed by 29 cycles of 30 sec at 94°C (denaturing), 30 sec at 52°C (annealing) and 60 sec at 72°C (elongation), with a final extension at 72°C for 10 minutes. Separate plates that contained negative controls without a template for each of the 96 barcoded primers were included for each set of plate processed, if one of these sample was positive, the samples and negative control plates were rerun with new primers. The presence of amplicons was confirmed by gel electrophoresis on a 2% agarose gel and stained with SYBRGreen (Ambion). PCR products were quantified using Quant-iT Picogreen® quantification system (Invitrogen) and equimolar amounts (100 ng) of the PCR amplicons were mixed in a single tube using the QIAgility robotic platform. Amplification primers and reaction buffer were removed by processing the amplicons’ mixture with the AMPure Kit (Agencourt). All PCR amplification reactions that failed were repeated twice using different amounts of template DNA and if these failed the samples were excluded from the analysis.

**Library preparation, sequencing read quality assessment, analysis and taxonomic assignments**

The purified amplicon mixtures were sequenced by 454 pyrosequencing using 454 Life Sciences® primer A by the Genomics Resource Center at the Institute for Genome Sciences, University of Maryland School of Medicine using Roche/454 Titanium chemistries and protocols recommended by the manufacturer and as amended by the Center.

In a first step, all sequences were trimmed before the first ambiguous base pair. The QIIME software package (version 1.6.0) [[7](#_ENREF_7)] was used for quality control of the remaining sequence reads using the split-library.pl script and the following criteria: 1) minimum and maximum length of 250 bp and 450 bp; 2) an average of q25 over a sliding window of 25 bp. If the read quality dropped below q25 it was trimmed at the first base pair of the window and then reassessed for length criteria; 4) a perfect match to a barcode sequence; 5) a match to *E. coli* 16S rRNA gene and 6) presence of the 534R 16S primer sequence used for amplification. Sequences were binned based on sample-specific barcode sequences and trimmed by removal of the barcode and primer sequences (forward if present and reverse). High quality sequence reads were first de-replicated using 99% similarity using the UCLUST software package [[8](#_ENREF_8)] and detection of potential chimeric sequences was performed using the UCHIME component of UCLUST [[9](#_ENREF_9)] with the *de novo* algorithm. Chimeric sequences were removed prior to taxonomic assignments.

Taxonomic assignments were performed as described by Ravel *et al.* [[4](#_ENREF_4)] using a combination of the pplacer and speciateIT (speciateIT.sourceforge.net). Taxonomic assignments (sequence read counts and relative abundances) are shown in **Additional File 3**.

**References**

1. Amsel R, Totten PA, Spiegel CA, Chen KC, Eschenbach D, Holmes KK: **Nonspecific vaginitis. Diagnostic criteria and microbial and epidemiologic associations**. *Am J Med* 1983, **74**(1):14-22.

2. Nugent RP, Krohn MA, Hillier SL: **Reliability of diagnosing bacterial vaginosis is improved by a standardized method of gram stain interpretation**. *J Clin Microbiol* 1991, **29**(2):297-301.

3. Forney LJ, Gajer P, Williams CJ, Schneider GM, Koenig SS, McCulle SL, Karlebach S, Brotman RM, Davis CC, Ault K *et al*: **Comparison of self-collected and physician-collected vaginal swabs for microbiome analysis**. *J Clin Microbiol* 2010, **48**(5):1741-1748.

4. Ravel J, Gajer P, Abdo Z, Schneider GM, McCulle SL, Koenig SSK, Karlebach S, Gorle R, Russell J, Tackett CO *et al*: **The vaginal microbiome of reproductive age women**. *Proc Nat Acad Sci* 2011, **108 .** (Suppl 1):4680-4687.

5. Frank JA, Reich CI, Sharma S, Weisbaum JS, Wilson BA, Olsen GJ: **Critical evaluation of two primers commonly used for amplification of bacterial 16S rRNA genes**. *Appl Environ Microbiol* 2008, **74**(8):2461-2470.

6. Consortium HMP: **Structure, function and diversity of the healthy human microbiome**. *Nature* 2012, **486**(7402):207-214.

7. Caporaso JG, Kuczynski J, Stombaugh J, Bittinger K, Bushman FD, Costello EK, Fierer N, Pena AG, Goodrich JK, Gordon JI *et al*: **QIIME allows analysis of high-throughput community sequencing data**. *Nature methods* 2010, **7**(5):335-336.

8. Edgar RC: **Search and clustering orders of magnitude faster than BLAST**. *Bioinformatics* 2010, **26**(19):2460-2461.

9. Edgar RC, Haas BJ, Clemente JC, Quince C, Knight R: **UCHIME improves sensitivity and speed of chimera detection**. *Bioinformatics* 2011, **27**(16):2194-2200.
