# Supplementary figures and images for: Daily temporal dynamics of vaginal microbiota before, during and after episodes of bacterial vaginosis
Source: Microbiome. 2013 Dec 2;1:29. doi: 10.1186/2049-2618-1-29 (PMC3968321; doi:10.1186/2049-2618-1-29)

**A.**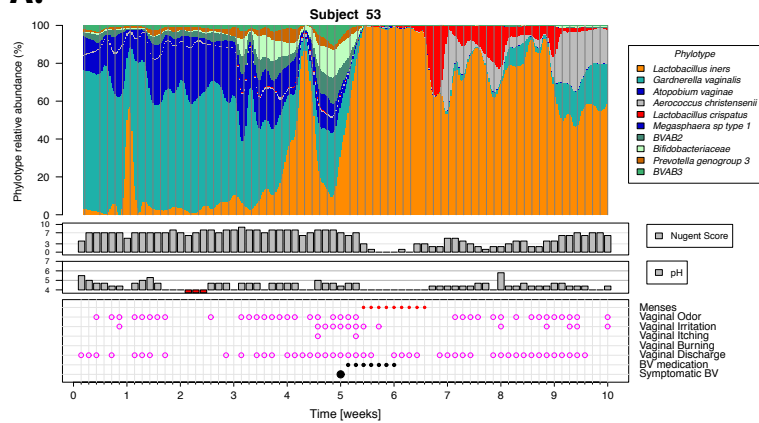**B.**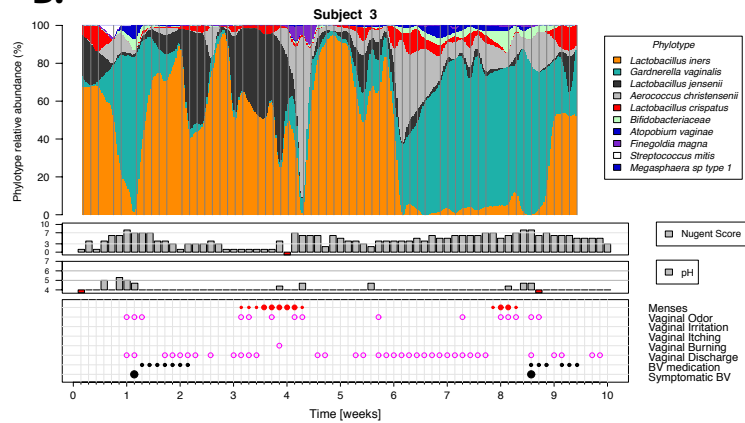**C.**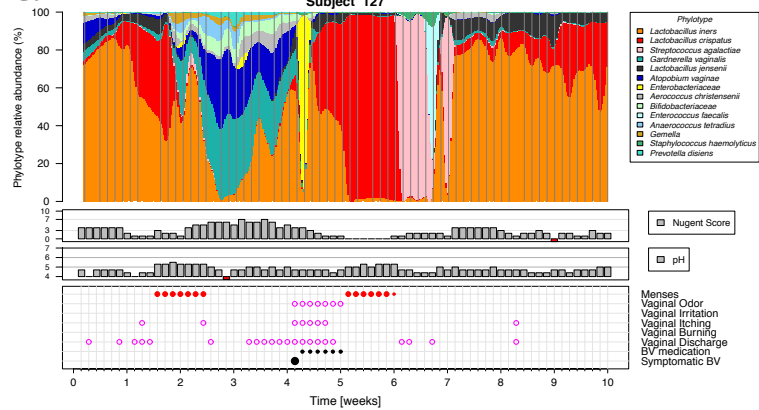**D.**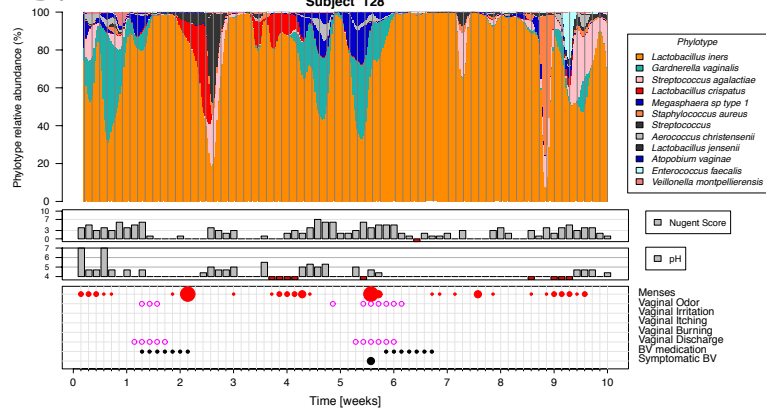**E.**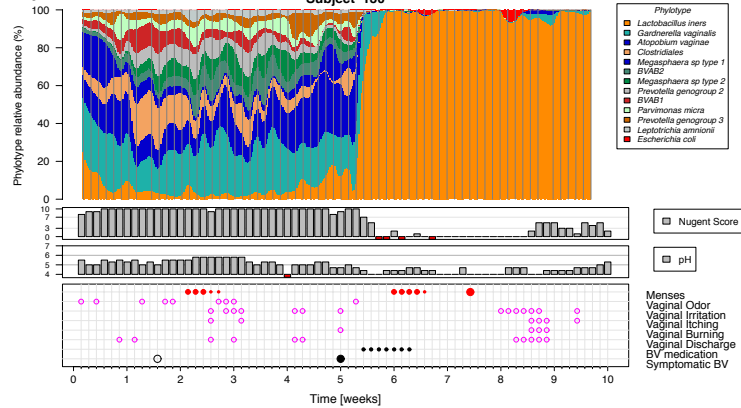**F.**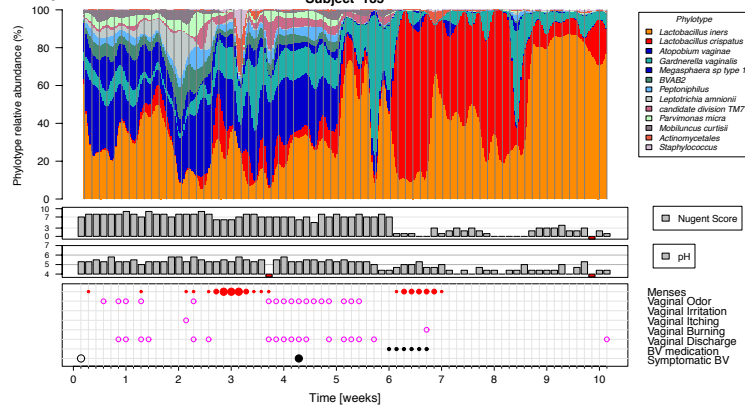**G.**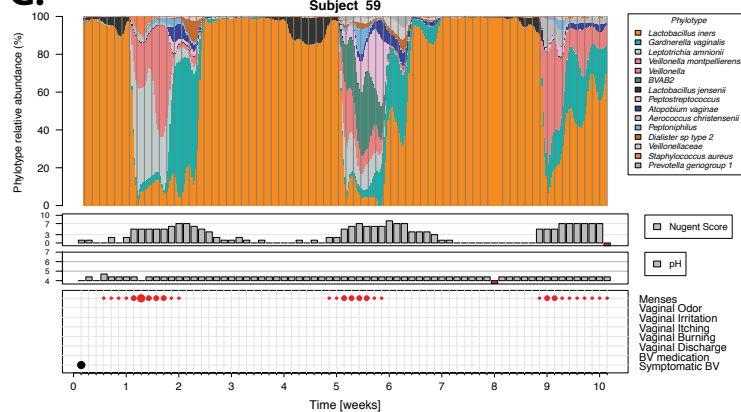**H.**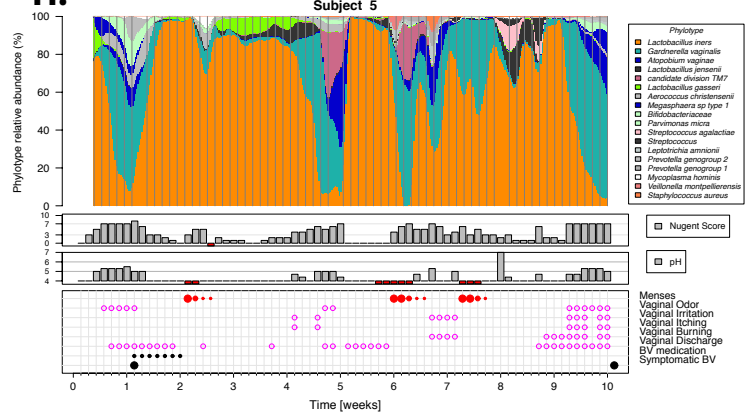

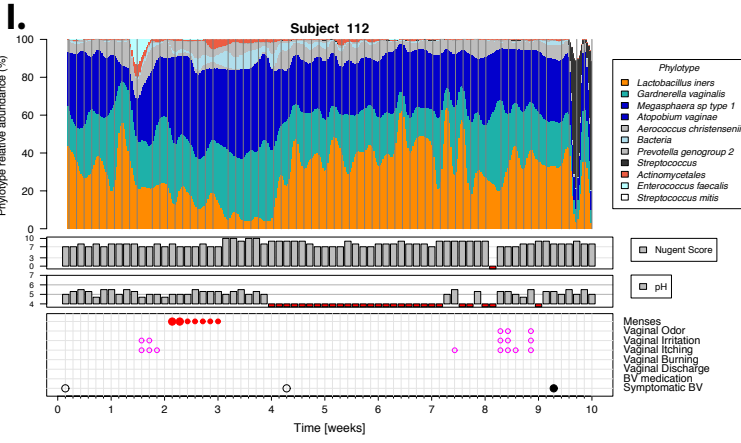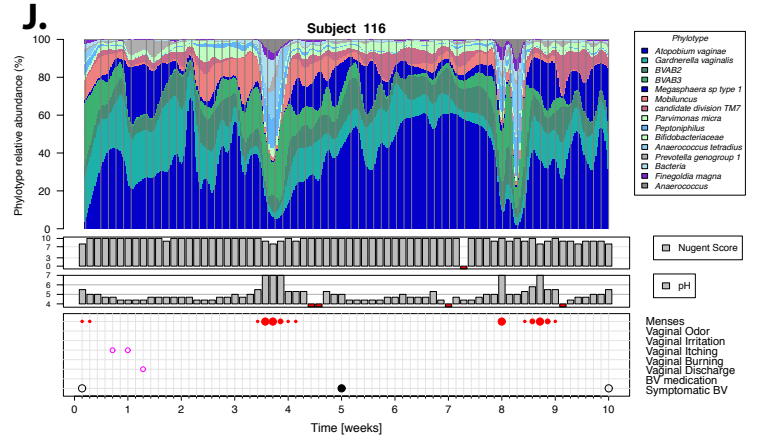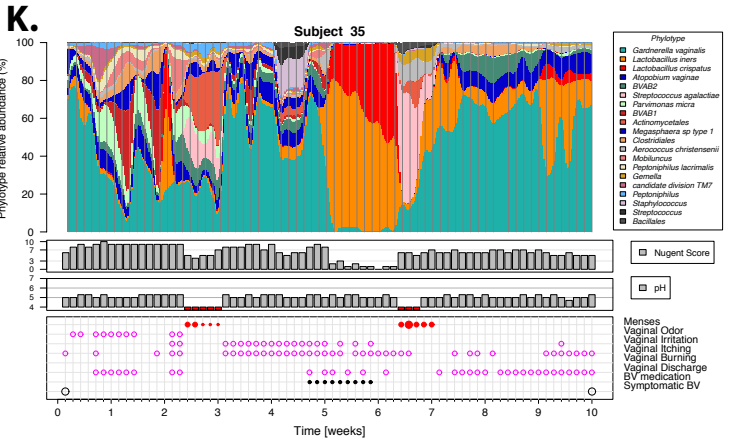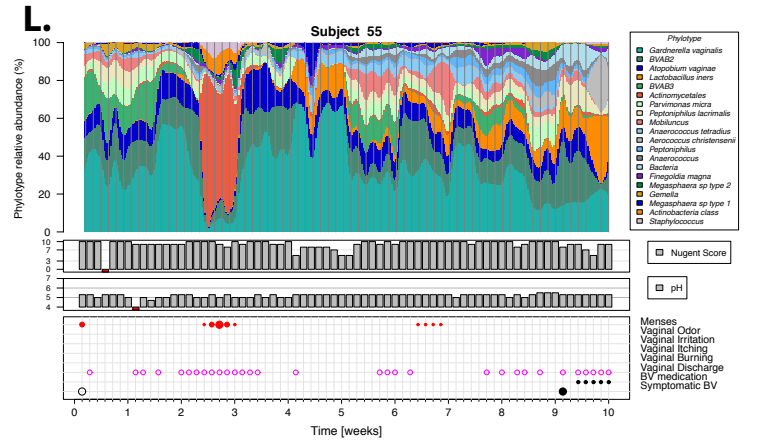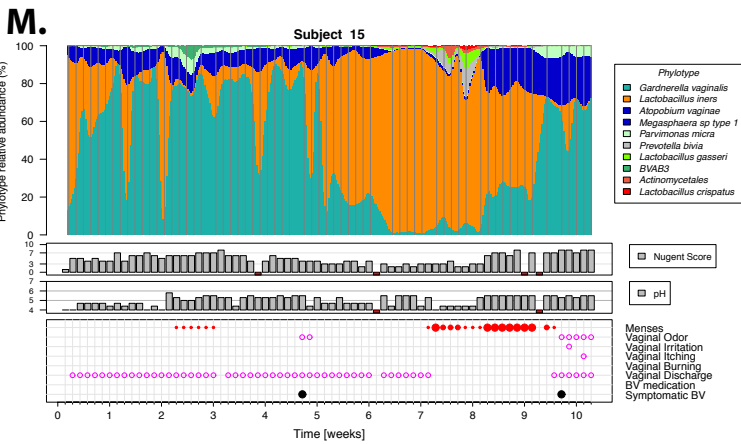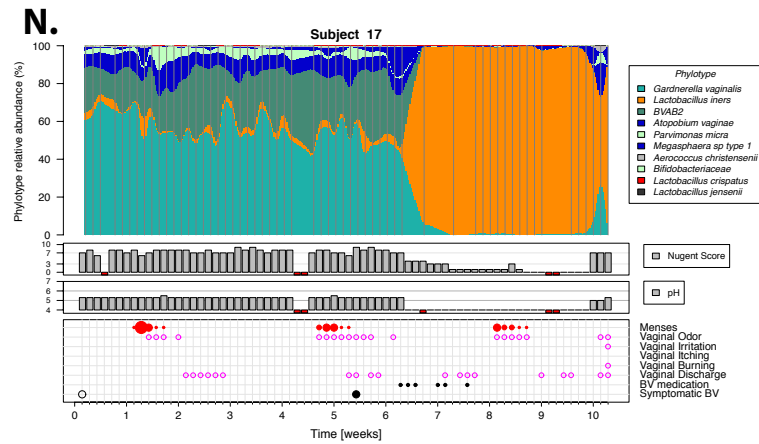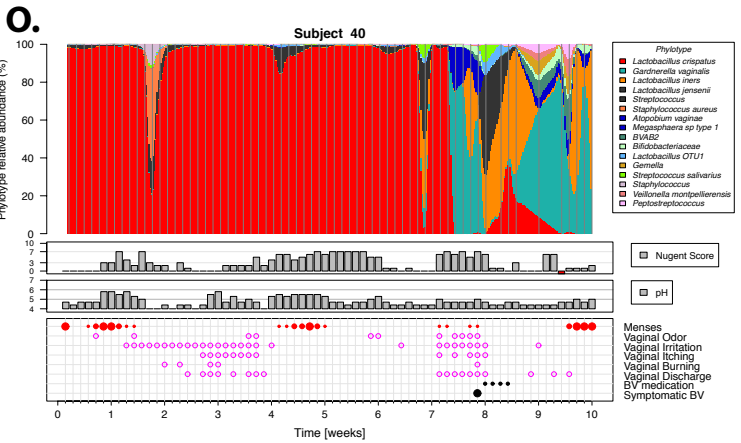

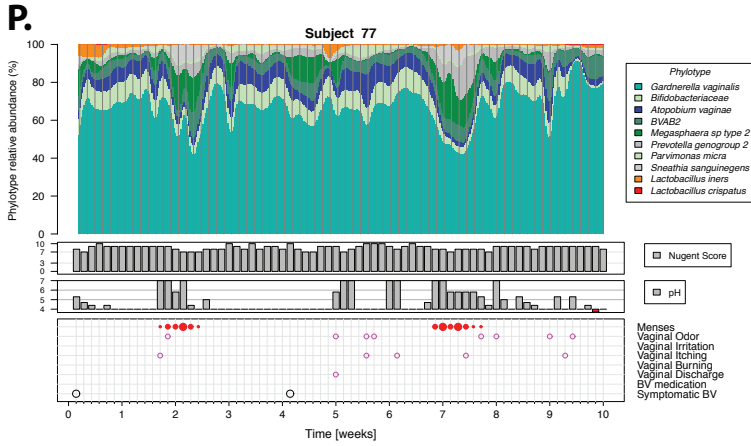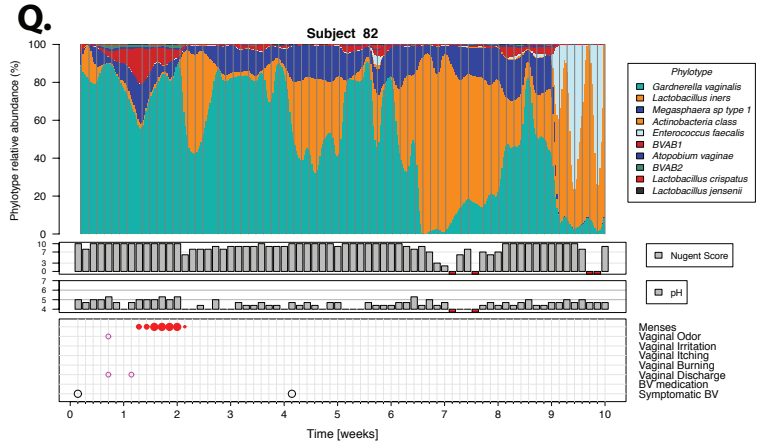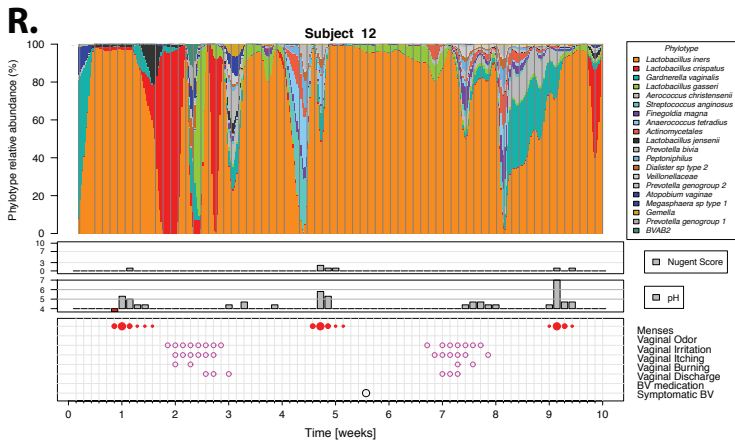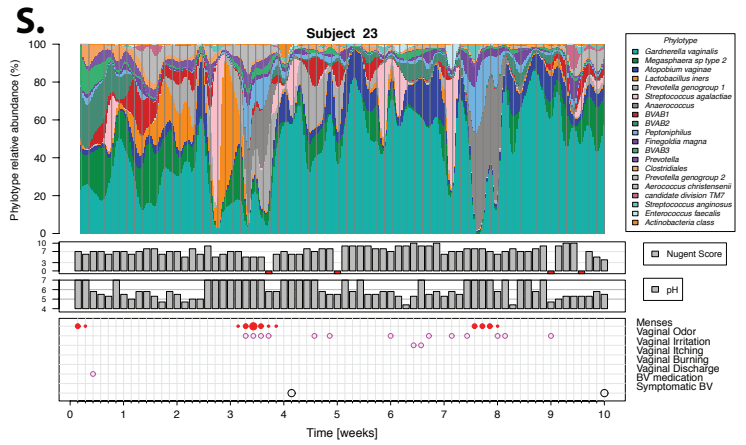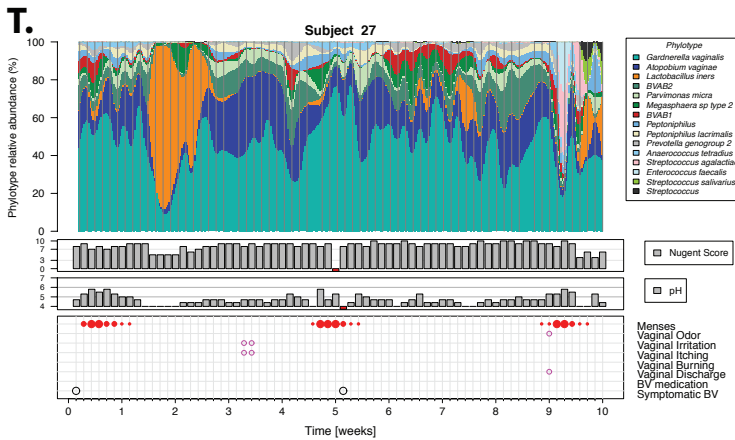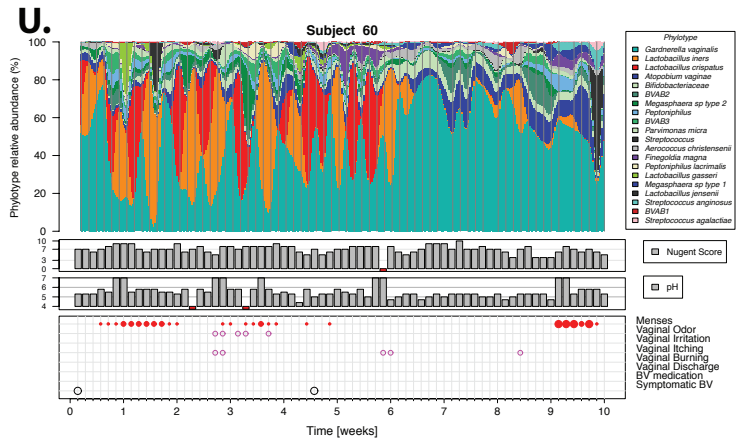

V.

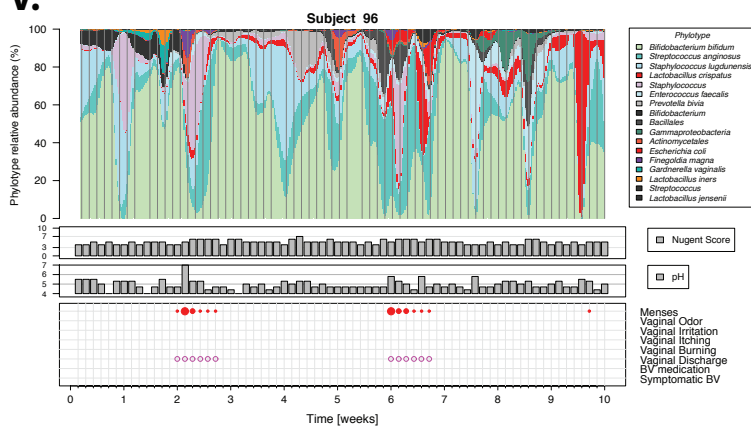

W.

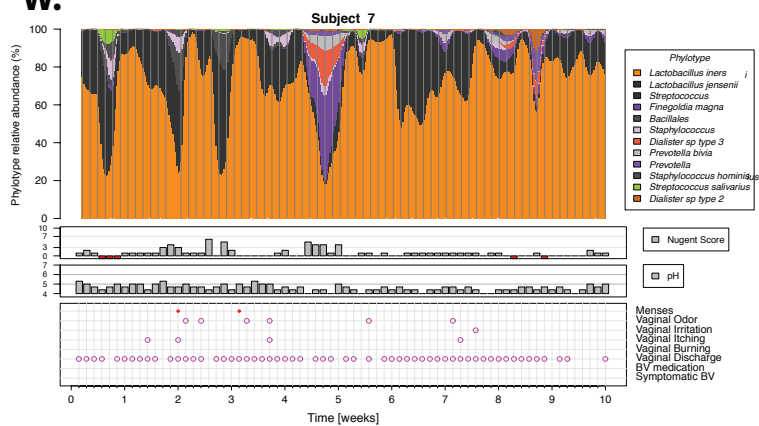

X.

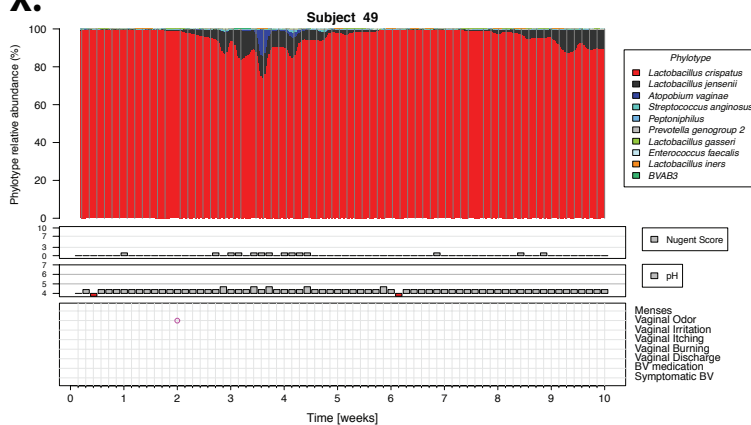

Y.

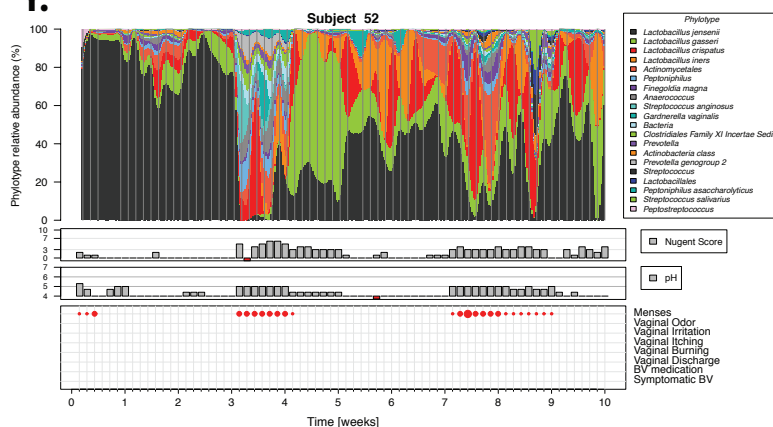

Supplement: Additional file 4: Figure S1 — Daily temporal dynamics of vaginal bacterial communities in 15 women who experienced symptomatic BV diagnosis (A to O), asymptomatic BV only (P to U) at some point during a 10-week period or no BV (V to Y). Color codes for each phylotype represented in the interpolated bar plots are shown next to each panel. Pink open circles: symptomology; red closed circles: menstruation days (small circles indicate low bleeding, medium circles, medium bleeding and large circles heavy bleeding); large black open circles: asymptomatic BV diagnosis; large black closed circles: symptomatic BV diagnosis; small closed circles: BV medication used. Daily Nugent scores (range 0–10) and pH (range 4–7) are indicated below the graph. [file 2049-2618-1-29-S4.pdf]
